# Europium-Doped Ceria Nanowires as Anode for Solid Oxide Fuel Cells

**DOI:** 10.3389/fchem.2020.00348

**Published:** 2020-05-25

**Authors:** Shuai Li, Xia Lu, Siqi Shi, Liquan Chen, Zhaoxiang Wang, Yusheng Zhao

**Affiliations:** ^1^Department of Physics, Academy for Advanced Interdisciplinary Studies, Southern University of Science and Technology, Shenzhen, China; ^2^Shenzhen Key Laboratory of Solid State Batteries, Guangdong Provincial Key Laboratory of Energy Materials for Electric Power, Southern University of Science and Technology, Shenzhen, China; ^3^Key Laboratory for Renewable Energy, Beijing Key Laboratory for New Energy Materials and Devices, Institute of Physics, Chinese Academy of Sciences, Beijing, China; ^4^School of Materials, Sun Yat-sen University, Guangzhou, China; ^5^School of Materials Science and Engineering, Shanghai University, Shanghai, China

**Keywords:** europium-doped ceria, oxygen vacancy, conductivity, anode, SOFC

## Abstract

CeO_2_-based materials have been studied intensively as anodes for intermediate temperature solid oxide fuel cells (IT-SOFCs). In this work, pristine and europium (Eu)-doped CeO_2_ nanowires were comprehensively investigated as anode materials for IT-SOFCs, by a combination of theoretical predictions and experimental characterizations. The results demonstrate: (1) Oxygen vacancies can be energetically favorably introduced into the CeO_2_ lattice by Eu doping; (2) The lattice parameter of the ceria increases linearly with the Eu content when it varies from 0 to 35 *mol*.%, simultaneously resulting in a significant increase in oxygen vacancies. The concentration of oxygen vacancies reaches its maximum at a ca. 10 *mol*.% Eu doping level and decreases thereafter; (3) The highest oxygen ion conductivity is achieved at a Eu content of 15 *mol*.%; while the 10 *mol*.% Eu-doped CeO_2_ sample displays the highest catalytic activity for H_2_-TPR and CO oxidization reactions. The conducting and catalytic properties benefit from the expanded lattice, the large amount of oxygen vacancies, the enhanced reactivity of surface oxygen and the promoted mobility of bulk oxygen ions. These results provide an avenue toward designing and optimizing CeO_2_ as a promising anode for SOFCs.

## Research Highlights

- Europium-doped ceria nanowires were synthesized; a maximum 35 *mol*.% Eu doping into the fluorite CeO_2_ lattice can be experimentally achieved.
- The oxygen vacancies were indicated by theoretical calculations, and quantitatively determined by Raman spectroscopy of the Eu-doped CeO_2_ samples.
- The 10 *mol*.% Eu-doped CeO_2_ has the highest content of oxygen vacancies, while the 15 *mol*.% Eu-doped CeO_2_ has the highest oxygen ion mobility.
- The 10 *mol*.% Eu-doped CeO_2_ sample was demonstrated to have the best catalytic activity for H_2_-TPR and CO conversion reactions.

## Introduction

Solid oxide fuel cells (SOFCs) are a kind of high-efficiency and environmental-friendly energy conversion device (Steele and Heinzel, 2001; Brandon et al., 2003; Ormerod, 2003; Choudhury et al., 2013). However, traditional SOFCs based on yttrium stabilized zirconium (YSZ) electrolytes have to work above 800°C due to the low conductivity of the electrolytes and the low catalytic activity of the electrodes (Jacobson, 2010; Coduri et al., 2018; Sreedhar et al., 2019). At such high temperatures, many materials are not stable. Therefore, it is essential to develop SOFC materials with higher conductivity and/or catalytic activity at lower working temperatures (Hibino et al., 2000; Brett et al., 2008; Wachsman and Lee, 2011). Recently, ceria related materials have been widely studied as electrode materials for intermediate temperature SOFCs (IT-SOFCs) for their unique conduction and catalytic properties at around 500–800°C (Marina et al., 1999; Murray et al., 1999; Goodenough, 2003; Al-Musa et al., 2015; Fan et al., 2017).

SOFCs require anodes that have high ionic (O^2−^) and electronic conductivities for quick charge transfer at the triple-phase-boundary (TPB) and a high catalytic activity for fuel gas oxidation (Jiang and Chan, 2004). Pristine CeO_2_ has a fluorite structure in the space group *Fm*-3*m*. Although fast oxygen ion migration is limited in a perfect CeO_2_ lattice due to a lack of intrinsic oxygen vacancies, CeO_2_ can be tuned to be an O^2−^ conductor or ion-electron mixed conductor by elemental doping (Mogensen et al., 2000; Skorodumova et al., 2002). On one hand, doping trivalent cations in CeO_2_ is an effective way to introduce oxygen vacancies into the lattice, and hence increases the oxygen ionic conductivity (Kim and Ann, 1989; Omar et al., 2007). Anderson et al. suggested that, in order to elevate the ionic conductivity of CeO_2_, the ideal effective atomic number of the dopant should be between 61 and 62 (Andersson et al., 2006). Sm^3+^ (*Z* = 62) and Gd^3+^ (*Z* = 63) doped CeO_2_ materials (SDC and GDC) were reported to demonstrate the highest oxygen ionic conductivity at ~1.5 ×10^−2^ S cm^−1^ at 700°C (Mogensen et al., 2000; Steele, 2000; Omar et al., 2007). On the other hand, electronic conductivity was also observed for CeO_2_ in a reductive atmosphere and/or at high temperatures (Mogensen et al., 2000). Doping mixed-valence elements into CeO_2_ can enhance both the ionic and electronic conductivities (Mogensen et al., 2000; Baral and Sankaranarayanan, 2010). Furthermore, the repeatability of Ce^4+^/Ce^3+^ redox cycling and the remarkable oxygen storage capability of ceria enhance the catalytic activity of ceria (Sharma et al., 2000), which can be enhanced further by structural manipulation and morphology modification. For example, Pr and Sn doping enhances the catalytic activity of CeO_2_ for H_2_ or CO oxidization (Xiao et al., 2009; Xian et al., 2013). Reduction of particle size has also been proved effective in improving the catalytic activity of the anode by shortening the ionic diffusion distance and increasing the contacting specific surface area, as is the case in peony-like CeO_2_, which was reported to have a high specific surface area and superior catalytic activity was achieved by doping or surface modification (Sun et al., 2006; Xian et al., 2011).

Europium (Eu, *Z* = 63) is a rare earth element between Sm and Gd. The radius of Eu^3+^ (1.07Å) is slightly larger than that of Ce^4+^ (0.97Å). Therefore, Eu could be a promising dopant for CeO_2_ when aiming to achieve high ionic conductivity with subtle lattice distortions. Shuk et al. reported that Ce_0.85_Eu_0.15_O_1.925−δ_ has a conductivity of around 2.6 ×10^−2^ S cm^−1^ with predominantly oxide-ion mobility in air (Shuk et al., 2000). While Baral et al. reported that the total activation energies of Ce_0.80_Eu_0.20_O_0.19−δ_ were 1.13 and 0.91 eV below and above 470°C, respectively, plausibly indicating different conducting mechanisms in different temperature ranges (Baral and Sankaranarayanan, 2010). On the basis of theoretical calculations, it was shown that the formation energy of oxygen vacancies varies regularly with the atomic number of the dopant in CeO_2_ (Andersson et al., 2006; Tang et al., 2010), except for the Eu-doped structure as discussed later. In addition, europium always demonstrates a mixed valence (Eu^2+^/Eu^3+^) nature. As a result, Eu-doping in CeO_2_ is expected to improve both the ionic and electronic conductivities and enhance the catalytic performance accordingly. This suggests an important implication that the conduction and catalytic mechanisms of Eu-doped CeO_2_ are influenced by oxygen vacancies, which needs further investigation.

In this work, Ce_1−x_*Eu*_x_O_2−δ_ (0 ≤ x ≤ 0.40; EDC) nanowires were prepared via a co-precipitation method. X-ray diffraction and Raman spectroscopy were employed to characterize the structural properties and oxygen vacancy formation of the as-prepared EDC samples. The dependence of conductivity on Eu doping content was tested by alternating current electrochemical impedance spectroscopy (AC-EIS). The catalytic activity of EDC was evaluated in H_2_-TPR and CO oxidation reactions. The formation of oxygen vacancies and their influence on the conducting and catalytic performances were revealed as follows.

## Experimental Section

### Synthesis

EDC nanoparticles were synthesized via a co-precipitation reaction using NH_4_HCO_3_ as the precipitant. Commercial Ce(NO_3_)_3_.6H_2_O (99%) and Eu(NO_3_)_3_·6H_2_O (99%) were dissolved in de-ionized water homogenously and then the solution was slowly dropped into an NH_4_HCO_3_ aqueous solution. The molar concentrations of the metal ions and the precipitant were 0.15 and 0.5 M, respectively, and the molar ratio of all metal ions and NH^4+^ was 1:10 during this process. The mixture was continuously stirred at 80°C for 1 h and then filtered. The precipitate was repeatedly washed using de-ionized water and ethanol, and then dried at 80°C. The precursor was ground and then calcinated in air at 700°C for 5 h. The obtained light yellow powders (and pellets thereof) were referred to as CeO_2_, EDC5, EDC10, EDC15, EDC20, EDC25, EDC30, EDC35, and EDC40, corresponding to the Eu molar ratio of 0, 5, 10, 15%, etc., respectively. Ce_0.8_Sm_0.2_O_2−δ_ (SDC20) and Ce_0.9_Gd_0.1_O_2−δ_ (GDC10) samples were also synthesized by the same procedure for comparison.

### Characterizations

The composition of the samples was determined using the inductively coupled plasma Atomic Emission Spectroscopic method (Thermo Electron Corporation). The morphology of the samples was characterized using a scanning electron microscope (SEM, Hitachi S4800). The structure of the powders was analyzed on an X-ray diffractometer (X'Pert Pro MPD) using Cu-Kα radiation (λ ~ 1.54053Å) and a JY HR800 Raman spectrometer (514 nm radiation and 2 cm^−1^ resolution). The XRD refinement was performed with FullProf software. The specific surface areas were measured by the multi-point BET method.

Each EDC powder was combined with a polyvinyl butyral binder and pressed into ϕ 10 ×1 mm pellets and sintered at 1,350°C for 10 h in air. After cooling down naturally, both sides of the pellets were polished smooth using Al_2_O_3_ paper. Silver paste was brushed uniformly on both sides of the pellet and then heated at 750°C for 0.5 h. Then EIS measurement was carried out on an IM6e electrochemical station in air (10 mV perturbation voltage and between 3 MHz and 100 mHz) and recorded as a function of temperature from 200 to 550°C. The reaction activity of the samples with H_2_ was measured on a temperature programmed desorption/reduction (TPD/TPR) instrument (TP-5,080). The EDC nanowires were pelletized to−40 ~ + 60 M, preheated in N_2_ at 400°C for 5 min and cooled down to room temperature, then heated to 900°C at a rate of 10°C min^−1^ in flowing N_2_ (*ca*. 30 ml min^−1^) containing 3 *vol*.% H_2_ for testing.

The catalytic activity of CO oxidation was tested under atmospheric pressure in a quartz tube reactor. Typically, 50 mg of catalyst was set in the middle of the quartz reactor using quartz wool. A continuous feed with a constant CO/O_2_/N_2_ at a volume ratio of 2:3:95 was used in this experiment. The effluent gas was analyzed using an on-line gas chromatograph (7890a Agilent) equipped with Porapak Q, ShiCarbon ST and a thermal conductivity detector. The test temperature was varied between 55 and 300°C, and measured at every 15°C interval. The conversion of CO was determined as χ_CO_ = [CO_2_]_out_ / ([CO]_out_ + [CO_2_]_out_).

### Theoretical Calculations

All total energy density functional theory (DFT) calculations were performed using the Vienna *ab initio* simulation package (VASP) in the framework of the projector augmented wave method. The Perdew-Burke-Ernzerhof functional was taken to deal with the exchange correlation potential. In order to account for the strong on-site Coulomb repulsion among the Ce 4*f* electrons, a Hubbard parameter *U* was added to the DFT functional (DFT+*U, U* = 4.5 eV). The details for the first-principle simulations are given in a previous work (Yang et al., 2006). The oxygen vacancy formation energy (*E*_vac_) was roughly estimated by: *E*_*vac*_ = *E(cell*_*vac*_*)* + $\frac{1}{2}$ *E(O*_2_*)* − *E(cell)*, where *E*(*cell*_*vac*_) and *E*(*cell*) are the total energies of the optimized supercells with and without an oxygen vacancy, respectively, and *E*(*O*_2_) is the total energy for the ground state of an optimized oxygen molecule in the gas phase. Note that, during the process of the oxygen vacancy formation, the released oxygen atom is expected to form an oxygen molecule.

## Results and Discussion

**Figure 1:**
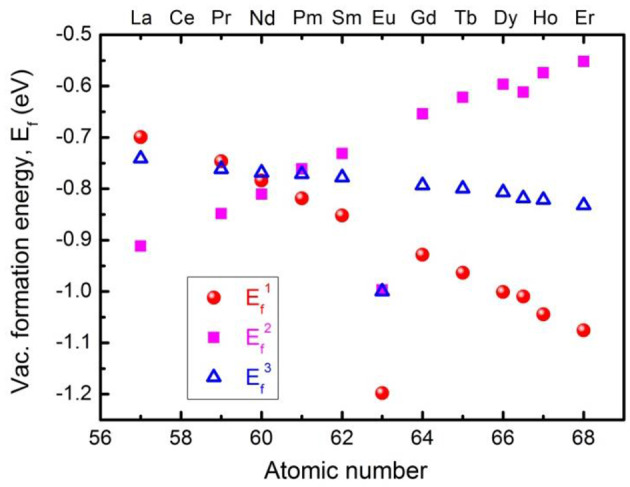
Formation energies of oxygen vacancy in low content (~3.1%) *M*-doped CeO_2_ (*M* = La, Pr, Nd, Pm, Sm, Eu, Gd, Tb, Dy, Ho, Er). The notations of $E_{f}^{1}$, $E_{f}^{2}$, and $E_{f}^{3}$ indicate that the oxygen vacancies are the first, second, and third nearest neighbors to the dopant ions, respectively.

Before the experimental synthesis and characterization of pristine and Eu-doped CeO_2_ samples, first-principle DFT simulations were performed to explore the formation mechanism and formation energy of oxygen vacancies in the fluorite CeO_2_ structure with/without dopants, which could then be used as a good guide for the subsequent experimental investigations. The derived formation energy, *E*_vac_, for pristine CeO_2_ was estimated to be 3.10 eV (Tang et al., 2010), which is very close to the results of 3.03 eV given by PBE+*U* (Yang et al., 2006), or 3.61 eV from LDA+*U* calculations (Andersson et al., 2006). When an oxygen vacancy (*V*_*O*_ in Kröger-Vink notation) is introduced into the CeO_2_ structure, two electrons will be localized at the 4*f* orbital of the Ce ions due to the strong correlation interaction, thus transforming two adjacent Ce^4+^ ions into a Ce^3+^ state and resulting in an obvious distortion of the CeO_6_ octahedron due to small-polaron behavior. Figure 1 demonstrates the formation energies of oxygen vacancies in low content (~3.1 *mol*.%) ***M***-doped CeO_2_ (***M***= La, Pr, Nd, Pm, Sm, Eu, Gd, Tb, Dy, Ho, and Er) structures. The results demonstrate that the formation energy of oxygen vacancies in CeO_2_ changes remarkably with different ***M***-dopants (the atomic number ranges from 57 to 68), indicating a modified structure with excellent oxygen storage and transport applications, *viz*. the existence of oxygen vacancies in the ***M***-doped CeO_2_ structures are more energetically favorable than in the pristine one. This provides insights into the material design and optimization of CeO_2_-based anodes for SOFCs. More specifically, the Eu-doped CeO_2_ (EDC) structure demonstrates the lowest oxygen vacancy formation energy of all the studied alternatives, as shown in Figure 1, which implies a promising route for research.

**Figure 2:**
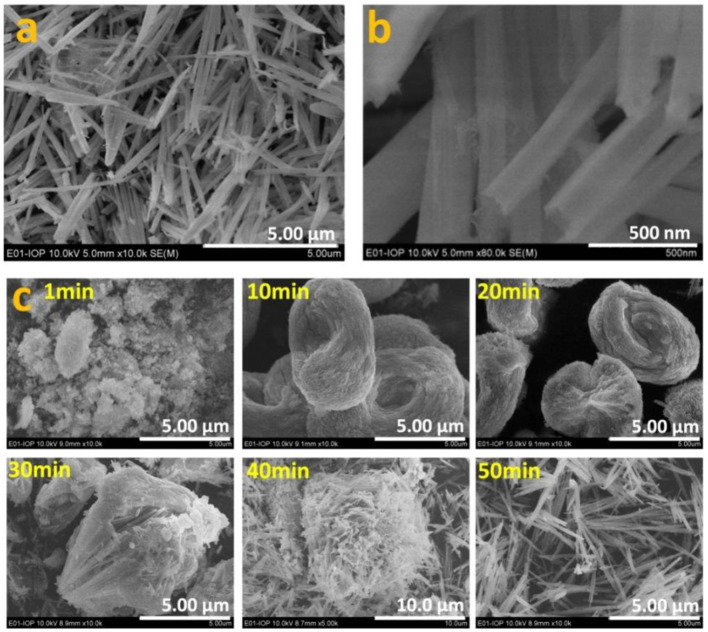
Morphology of the calcinated EDC5 nanowires **(a)** and the magnified image **(b)**. The morphology evolution of the precursor with aging time during the co-precipitation process **(c)**.

As well as the theoretical investigation into oxygen vacancy formation energy, Eu-doped CeO_2_ samples were synthesized and characterized systematically. Figures 2a,b show that the 5 *mol*.% Eu-doped CeO_2_ powder is composed of uniform nanowires of 5–10 μm in length and 100 nm in width. The nanowires are actually formed during the co-precipitation process (Figure 2c). It is interesting that the co-precipitated precursor is initially nanoparticles and then aggregates into larger nest-like clusters. The nest-like nanostructure is finally disintegrated into nanowires as the aging time increases to *ca*. 50 min (Figure 2c). Calcination at 700°C does not change the morphology of the co-precipitates (Figure S1). CeO_2_ nanowires with other Eu doping levels (Ce_1−x_*Eu*_x_O_2−δ_; 0 ≤ x ≤ 0.20) can be obtained under the same conditions. The width of the EDC nanowires decreases gradually with increasing Eu content. At even higher Eu doping levels (0.25 ≤ x ≤ 0.40), uniform nanoparticles of 10–20 nm are obtained (Figure S1). The morphology of the co-precipitation precursor remain unchanged after high temperature calcinated (Figure S2). In addition, the specific area of the EDC powders is *ca*. 30 m^2^/g, and is roughly independent of the Eu doping content.

**Figure 3:**
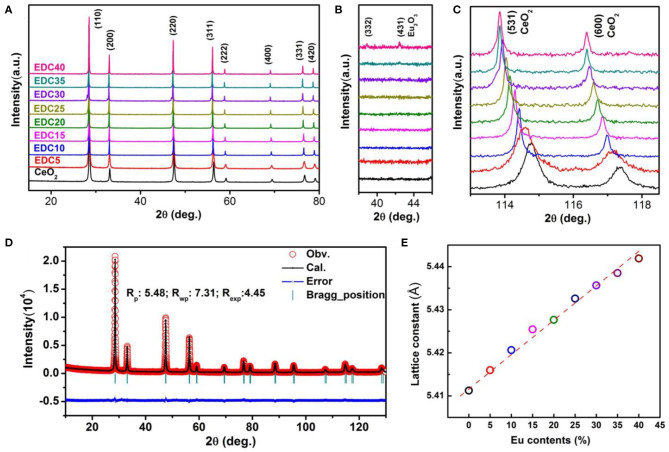
XRD patterns of the Eu-doped ceria **(A)**. Impurity phase (Eu_2_O_3_) is not observed until the Eu content is at 40 *mol*.% (EDC40) **(B)**. The change in the diffraction peaks of CeO_2_ upon Eu doping at high diffraction angles **(C)**. A typical refined XRD pattern of EDC nanowires (EDC5) **(D)**, with the experimental data (red circles), calculated pattern (black line), Bragg positions (green markers), and difference curve (blue line). The refinement was done using FullProf software on the basis of the *Fm*-3*m* space group. The lattice parameters of EDC varies with Eu contents **(E)**.

The XRD patterns of the as-synthesized EDC samples with different Eu content are shown in Figure 3. It can be seen clearly that all the XRD patterns of the EDC samples can be well-indexed into the fluorite structure. The Miller indices of the main peaks have been marked in Figure 3A. In addition, the calcinated nanoparticles and the calcinated nanowires show the same XRD patterns as long as their Eu doping content is the same (Figure S1). There are no detectable impure phases in the CeO_2_ samples with Eu molar content ranging from 0 to 35%. A diffraction peak for the Eu_2_O_3_ (431) plane is detected in the EDC40 sample as shown in the magnified Figure 3B. As the Eu content increases, the main peaks of CeO_2_ shift gradually toward a lower angle, as is shown for the magnified CeO_2_ (531) and (660) diffractions in Figure 3C. Particle size of the as-synthesized EDC samples ranges from 10 to 20 nm, calculated by the Scherrer Formula. To obtain the lattice parameters of EDC, refinement was performed using FullProf software. Figure 3D shows the typical refinement results of the EDC5 sample. The lattice constant is 5.411Å for the pristine CeO_2_ and increases linearly with the molar content of Eu, obeying Vegard's rule (Denton and Ashcroft, 1991) (Figure 3E and Table S1). These results indicate that Eu is successfully doped into CeO_2_ with solid solubility *ca*. 35 *mol*.%. As a matter of fact, substitution of larger Eu^3+^ ions for smaller Ce^4+^ ions in the CeO_2_ lattice results in lattice distortion and the generation of oxygen vacancies for charge balance. Additionally, it is notable that the diffraction peaks narrow gradually as the Eu doping content increases, revealing better crystallinity of the CeO_2_ samples with higher Eu doping levels.

**Figure 4:**
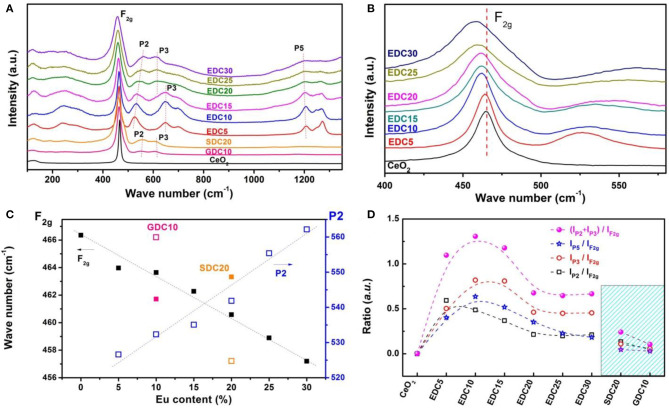
Raman spectra of Eu-doped ceria **(A)** and the enlarged Raman spectra (400–550 cm^−1^) **(B)**, peak shifts around 460 cm^−1^ (solid black square) and 550 cm^−1^ (blue rectangles) of EDC, SDC20 (orange), and GDC10 (pink) **(C)**. The ratio of integrated intensity of peaks P2 (*ca*. 550 cm^−1^) and F_2g_ (*ca*. 460 cm^−1^) (black rectangles, I_P2_/I_F2g_), P3 (*ca*. 640 cm^−1^) and F_2g_ (red circles, I_P3_/I_F2g_), P5 (*ca*. 1,200 cm^−1^) and F_2g_ (blue stars, I_P5_/I_F2g_), and the total effect [pink solid circles (I_P2_ + I_P3_ + I_P5_)/I_F2g_], respectively **(D)**.

However, the XRD pattern is insensitive to the oxygen vacancies, since they are highly dispersive and of low concentration. To get further information about the oxygen vacancies, the Raman spectra of EDC samples was collected. The Raman spectrum of SDC and GDC was also collected for comparison (Figure 4A). It can be unambiguously seen that the shape of the spectrum changes gradually with the Eu doping level. In the Raman spectrum of CeO_2_, there is a strong band located at around 465 cm^−1^ noted as F_2g_. According to previous reports (Weber et al., 1993; Mcbride et al., 1994; Pushkarev et al., 2004), this is the only Raman-active mode with triple-degenerated F_2g_ symmetry in a perfect CeO_2_ lattice, which can be viewed as a symmetrical breathing mode of the O atoms around the Ce^4+^ ions. In our case, the F_2g_ mode of pristine CeO_2_ is centered at 466 cm^−1^. It gradually shifts to lower frequencies and broadens as the Eu doping amount increases, associating with an asymmetrical trail at the low frequency side and a broad shoulder at the high frequency side (Figures 4A,B). The red-shifts of the F_2g_ mode indicate the Ce–O bond changes because of oxygen vacancy formation and lattice dilation as a result of Eu doping. The asymmetrical trail is a result of inhomogeneous strain in the Eu-doped CeO_2_ nanoparticles (Hernandez et al., 2010). In the spectra of doped CeO_2_ samples, a broad shoulder feature appears at around 550 cm^−1^ (denoted as P2), which has been attributed to the formation of oxygen vacancies by low valence cation doping (Weber et al., 1993; Mcbride et al., 1994; Pushkarev et al., 2004). The P2 feature, distinct from the F_2g_ mode, appears in the spectra of trivalent cation doped CeO_2_ samples (e.g., La^3+^, Pr^3+^, Nd^3+^, etc.), indicating the defect space (involving dopants and oxygen vacancies) deviates from a perfect MO_8_-type (each cation with 8 nearest O) (Mcbride et al., 1994; Fu et al., 2003; Dohcevic-Mitrovic et al., 2006). In this work, the P2 feature is prominent when the Eu content is 5 *mol*.%, and shifts to higher energies as the Eu concentration increases. Figure 4C clearly shows the F_2g_ mode (at 466 cm^−1^ for *x* = 0 and 457 cm^−1^ for *x* = 0.30) red-shifts and the P2 mode (at 526 cm^−1^ for *x* = 0.05 and 562 cm^−1^ for *x* = 0.30) blue-shifts in the Ce_1−x_Eu_x_O_2−δ_ samples. These features of SDC20 and GDC10 samples are also shown for comparison. These results distinctly confirm that Eu is successfully doped into the CeO_2_ lattice with oxygen vacancies introduced to compensate for the charge, and the structure distortion is noted as a linear expansion referring to the XRD analyses.

Besides the P2 feature, other peaks also appear in the Raman spectra of doped CeO_2_ samples. The peak noted as P3 (*ca*. 640 cm^−1^) is attributed to the oxygen vacancies related to Ce^3+^ in the CeO_2_ lattice (Pu et al., 2007). This feature is absent in the Raman spectrum of pristine CeO_2_, suggesting negligible intrinsic oxygen vacancies in the pristine ceria. It emerges with Eu-, Sm-, and Gd-doping, and becomes remarkable for EDC5, EDC10, and EDC15 samples. The peak noted as P5 (*ca*. 1,200 cm^−1^) is attributed to the O–O vibration in superoxide species, which comes from O_2_ adsorption on surface defects or oxygen vacancies in the surface or subsurface of Eu-doped CeO_2_ samples. As the P2, P3, and P5 modes are related to oxygen vacancies, and the F_2g_ mode indicates the Ce–O breathing mode, the ratio between integrated intensities of the P2, P3, P5 and that of the F_2g_ peaks (I_P2_/I_F2g_, I_P3_/I_F2g_, I_P5_/I_F2g_ (I_P2_ + I_P3_ + I_P5_)/I_F2g_) can be used to evaluate the concentration of oxygen vacancies (Mcbride et al., 1994). That is, the higher the ratio, the more oxygen vacancies in the lattice. Figure 4D shows the dependence of I_P2_/I_F2g_, I_P3_/I_F2g_, I_P5_/I_F2g_, and (I_P2_ + I_P3_ + I_P5_)/I_F2g_ on the Eu concentration in comparison with those of SDC20 and GDC10. The trend of I_P2_/I_F2g_ suggests that substitution of Ce^4+^ with Eu^3+^ can greatly enhance the amount of oxygen vacancies even with 5 *mol*.% Eu content; while this type of oxygen vacancy decreases gradually as the Eu concentration further increases. Meanwhile doping Eu^3+^ into CeO_2_ also results in the formation of Ce^3+^ and oxygen vacancies in the lattice, as indicated by the I_P3_/I_F2g_ ratio change, and this type of oxygen vacancy reaches its maximum amount at around the 10–15 *mol*.% doping level and then decreases gradually. For the surface and subsurface oxygen species, they are enhanced greatly by Eu^3+^ doping and reach a maximum around the 10 *mol*.% doping level as shown by the change in the I_P5_/I_F2g_ ratio in Figure 4D. In summary, Eu^3+^ doping into CeO_2_ introduces a large amount of oxygen vacancies in the lattice via charge balancing, and also enhances the surface O_2_ adsorption property. A possible reason could be that the ionic radius of Eu^3+^ is 1.07Å, a little bit larger than that of Ce^4+^ at 0.97Å. This makes it easy for Eu to enter into the CeO_2_ lattice and introduce many oxygen vacancies with little lattice distortion. Among all Eu-, Sm-, and Gd-doped CeO_2_ samples studied here, the optimal doping level is demonstrated to be around 10 *mol*.% Eu content to achieve the most oxygen vacancies.

**Figure 5:**
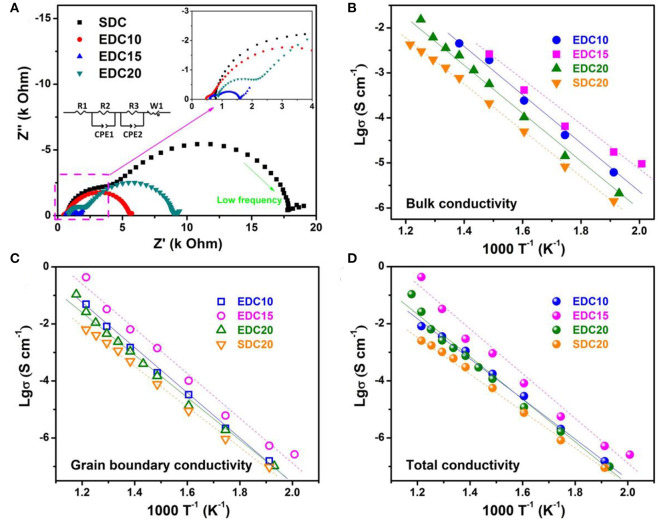
Impedance spectra of SDC, EDC10, EDC15, and EDC20 measured at 400°C in air; insets are the magnified spectra at a high frequency range and the equivalent circuit used for fitting **(A)**. Arrhenius plots of the bulk **(B)**, grain boundary **(C)**, and total **(D)** conductivities of the samples between 200 and 550°C.

Raman spectrum analysis confirms the introduction of oxygen vacancies into the Eu-doped CeO_2_ lattice, which is a prerequisite for better electrochemical performance of EDC as an anode. Furthermore, it is also important to investigate oxygen/vacancy diffusion kinetics. Here, alternating current electrochemical impedance spectroscopy (AC-EIS) was employed to investigate the Eu-content dependent conductivity of EDC10, EDC15, EDC20, and SDC20 samples. The pellets were sintered at 1,350°C for 10 h to get a dense ceramic, in an attempt to diminish the grain boundary resistance (details shown in Figure S3). The typical impedance spectra of these samples measured at 400°C in air (Figure 5A) consists of two typical semicircles. The semicircles at the higher frequency range (inset of Figure 5A) are ascribed to the bulk resistance. The semicircles at the lower frequency range are ascribed to grain boundary resistance. Equivalent circuit (inset of Figure 5A) is used to resolve the EIS spectrum. The variation of the calculated conductivities of the bulk, the grain boundary and the total are shown in Figures 5B–D and Table S2, respectively. They all basically obey the Arrhenius equation in the studied temperature range (200–550°C). The EDC15 sample has higher bulk, grain boundary and total conductivities than any other sample. At 550°C, the total conductivity of the EDC15 sample is 3.6 ×10^−1^ S cm^−1^, about two orders of magnitude higher than that of the SDC20 sample, one order higher than that of the EDC20 and EDC10 samples. Derived from the Arrhenius plot, the total conducting activation energy of EDC15 at the measured temperature range is 1.37 eV. Referring to above analysis, the enhanced conductivity probably arises from the higher concentration of oxygen vacancies in EDC15 than in the SDC20 and EDC20 samples. However, it is interesting that although the EDC10 sample has more oxygen vacancies, its conductivity is lower than that of the EDC15 sample. This could be attributed to the competitive effects of oxygen vacancy formation and migration. On one hand, in the EDC10 sample, it is probably easy for the oxygen vacancies to associate with surface/bulk oxygen species (as indicated by I_P5_/I_F2g_ in Figure 4D). On the other hand, the lattice constant of the EDC15 sample is larger than that of EDC10, favoring O^2−^ migration.

**Figure 6:**
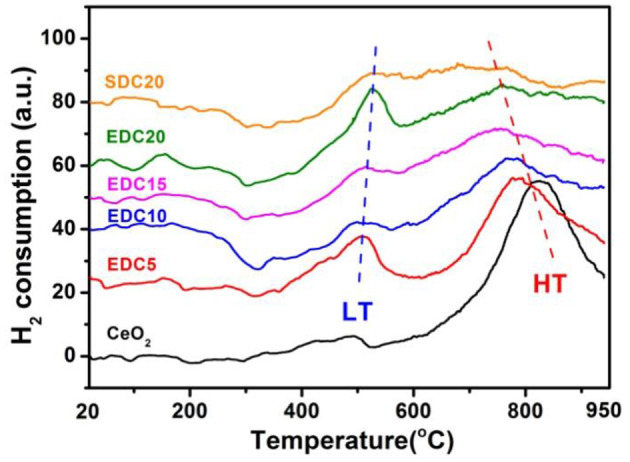
H_2_-TPR profiles of EDC nanowire samples with various Eu contents.

The morphology, grain size, lattice parameters, and ion migration have a large influence on catalytic properties (Zhao et al., 2018; Lian et al., 2019). Subsequently, improvement of the catalytic properties of the EDC samples was evaluated by H_2_-TPR and CO oxidation reactions. The reducibility of the pristine and EDC samples was examined by H_2_-TPR analysis as shown in Figure 6. The peak position and peak area in the H_2_-TPR profiles were determined as shown in Table 1. According to a previous report, the low temperature peak (LT, below 600°C) and the high temperature peak (HT, around 800°C) are ascribed to the reduction of the oxygen species on the surface and in the bulk of CeO_2_, respectively (Hernandez et al., 2010). In this work, the weak LT and strong HT peaks of pristine CeO_2_ nanowires are centered at 495 and 825°C, respectively. Doping Eu into CeO_2_ introduces abundant oxygen vacancies into the lattice, which changes the oxygen mobility and adsorption properties, and hence affects the reactivity of the LT and HT reduction processes. In more detail, firstly, the LT peak becomes prominent for Eu-doped CeO_2_ with more hydrogen consumption indicated by the integrated peak area as shown in Table 1. Secondly, the position of the LT peak shifts slightly to higher temperatures with increasing Eu content as shown in Figure 6. Thirdly, the HT peak shifts to lower temperatures, and the area of this peak becomes smaller, as the Eu content increases as shown in Figure 6 and Table 1. This is indicative of the enhanced oxygen mobility in the bulk with Eu-doping. However, the low reaction activity of surface oxygen and poor oxygen ion diffusion kinetics indicates that there are few oxygen vacancies in the pristine CeO_2_ sample, consistent with the Raman results as shown in Figure 4.

**Table 1:** H_2_-TPR results for EDC samples.

**Sample**	**Peak position (^**°**^C)**	**Peak area (a.u.)**		
		**(H**_****2****_ **consumption)**		
	**LT**	**HT**	**LT**	**HT**
CeO_2_	495	825	0.36	5.15
EDC5	510	781	1.61	3.68
EDC10	510	764	0.38	2.3
EDC15	515	761	0.51	2.22
EDC20	525	756	1.20	1.55

The color of the pure CeO_2_ sample is pale yellow due to Ce^4+^-O^2−^ charge transfer. It turns blue upon reduction (CeO_2−x_), and turns black when CeO_2_ becomes grossly non-stoichiometric (from over reduction) (Mogensen et al., 2000). However, it should be pointed out that in this work the color of CeO_2_ (pale yellow) became lighter as more Eu was doped. After H_2_-TPR testing and cooling down to room temperature, the pristine CeO_2_ and all of the doped CeO_2_ samples in the quartz tube become dark blue, indicating considerable reduction by H_2_ and formation of a non-stoichiometric CeO_2−x_ state with plenty of oxygen vacancies. However, the color of the reduced EDC10 sample recovered to yellow as soon as it was exposed to air, and even set fire to the weighing paper. Obviously this is an exothermic process, during which the H_2_-reduced sample regains oxygen from the air. The color change of the reduced EDC10 sample is the most prominent and strongest of all, with all of the particles turning back to yellow in a few seconds. For the H_2_-reduced EDC5, EDC15, EDC20, and SDC20 samples, the color change process is much slower and some of the particles remained dark blue in air. This color conversion phenomena implies that the 10 *mol*.% Eu doped CeO_2_ probably has the most stable fluorite structure with respect to other samples, and it also favors O^2−^ migration and O_2_ adsorption. In addition, nearly all the particles of the reduced CeO_2_ remained dark blue after exposure to air for a few days. These results suggest that Eu doping in the lattice significantly enhances the catalytic activity of CeO_2_ on H_2_-TPR. The catalytic performance demonstrates a Eu-content dependent relationship. Optimal catalytic activity is obtained when the doping level is 10 *mol*.% as shown by the H_2_-TPR tests above.

**Figure 7:**
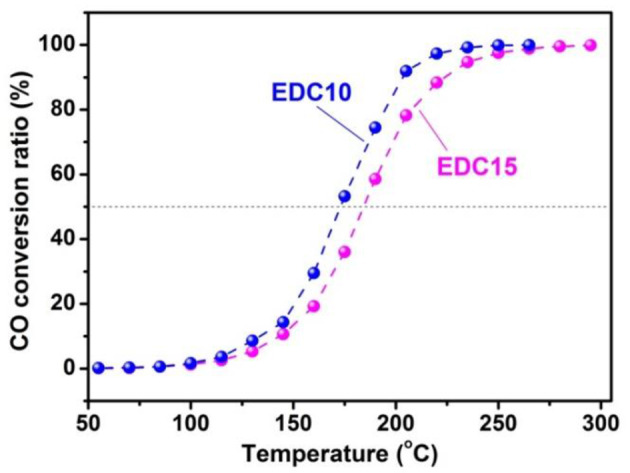
Catalytic activities of EDC10 and EDC15 nanowires for CO oxidation.

Concerning the real life conditions for use as an anode for SOFCs, the catalytic activity of the EDC10 (highest catalytic activity on H2-TPR) and EDC15 (highest conductivity) samples are further examined on CO oxidation reaction. The catalytic curves for EDC10 and EDC15 samples are shown in Figure 7. A CO conversion ratio of 100% is achieved before 300°C, indicating that both the EDC10 and EDC15 samples are promising candidates for anode materials. As predicted with the Raman spectroscopic analysis and H_2_-TPR test, the catalytic activity of EDC10 is found to be higher than that of EDC15 for CO oxidation. Figure 7 shows clearly that the T_50_ (temperature for 50% CO conversion) for EDC10 and EDC15 are 173 and 184°C, respectively, and T_90_ (temperature for 90% CO conversion) for EDC10 and EDC15 are 204 and 225°C, respectively. These results indicate that EDC10 can work at lower temperatures than EDC15 as an anode material for IT-SOFCs, and the catalytic performance of EDC10 is better accordingly. Since the samples under investigation have similar morphology and specific surface area, the difference in catalytic activity here is taken to arise from the comprehensive effects of oxygen vacancy concentration, oxygen ion mobility and reactivity as discussed above. This also confirms that the optimal content is around 10 *mol*.% Eu doping in CeO_2_ for application as an SOFC anode.

## Conclusions

Fluorite structure Eu-doped CeO_2_ samples, with nanowire morphology, were synthesized via a co-precipitation method. The solid solubility of Eu in CeO_2_ structures is *ca*. 35 *mol*.%. Below the solid solubility, the lattice parameter of ceria increases linearly with the content of Eu, obeying Vegard's law. Doping Eu into CeO_2_ introduces a large amount of oxygen vacancies, and hence enhances the conductivity and catalytic activity. Consequently, the EIS results show that EDC15 (15 *mol*.% Eu-doped CeO_2_) has the highest conductivity among the samples, while the H_2_-TPR and CO oxidation results demonstrate the EDC10 sample (10 *mol*.% Eu-doped CeO_2_) has the highest catalytic activity. The enhanced conducting and catalytic properties are ascribed to the formation of abundant oxygen vacancies, associated with the enhanced reactivity of surface oxygen and the mobility of bulk oxygen ions in the expanded lattice. Overall, Eu-doped CeO_2_ nanowires, especially the *ca*. 10 *mol*.% Eu doped sample, exhibit promising application potential as anode materials in IT-SOFCs. All of these findings provide a comprehensive path for material design and optimization for SOFCs.

## Author Contributions

SL contributed ideas, designed the work, performed experiments and data analysis, and wrote the manuscript. XL contributed to lattice parameter refinement. SS contributed to the theoretical calculations. LC, ZW, and YZ contributed to discussing this work, editing the manuscript, providing laboratory platform and financial support.

## Conflict of Interest

The authors declare that the research was conducted in the absence of any commercial or financial relationships that could be construed as a potential conflict of interest. The reviewer KZ declared a shared affiliation, though no other collaboration, with one of the authors SS to the handling editor.
