# Supplementary material for: Europium-Doped Ceria Nanowires as Anode for Solid Oxide Fuel Cells
Source: Front Chem. 2020 May 25;8:348. doi: 10.3389/fchem.2020.00348 (PMC7261932; doi:10.3389/fchem.2020.00348)
Supplement: Supplementary file 1 [file Data_Sheet_1.pdf]

Supplementary Material

Europium-doped Ceria Nanowires as Anode for Solid Oxide Fuel Cells

Shuai Li[1,2,3*], Xia Lu[4], Siqi Shi[5], Liquan Chen[3], Zhaoxiang Wang[3*], Yusheng Zhao[1,2*]

[1] Department of Physics, Academy for Advanced Interdisciplinary Studies, Southern University of Science and Technology, Shenzhen 518055, China

[2]Guangdong Provincial Key Laboratory of Energy Materials for Electric Power, Shenzhen 518055, China

[3]Key Laboratory for Renewable Energy, Chinese Academy of Sciences, Beijing Key Laboratory for New Energy Materials and Devices, Institute of Physics, Chinese Academy of Sciences, Beijing, 100190, China

[4]School of Materials, Sun Yat-sen University, Guangzhou, 510006, China

[5]School of Materials Science and Engineering, Shanghai University, Shanghai 200444, China

*** Correspondence:**
Dr. Shuai Li, Email: lis6@sustech.edu.cn; Prof. Zhaoxiang Wang, Email: zxwang@iphy.ac.cn; Prof. Yusheng Zhao, Email: zhaoys@sustech.edu.cn;

The morphology of the Eu-doped CeO_2 nanomaterials is formed during co-precipitation process, and remain the same after being calcinated at 700 ^oC in air. For the EDC samples with the same composition, the XRD pattern of the calcinated nanoparticles and nanowires are the same.

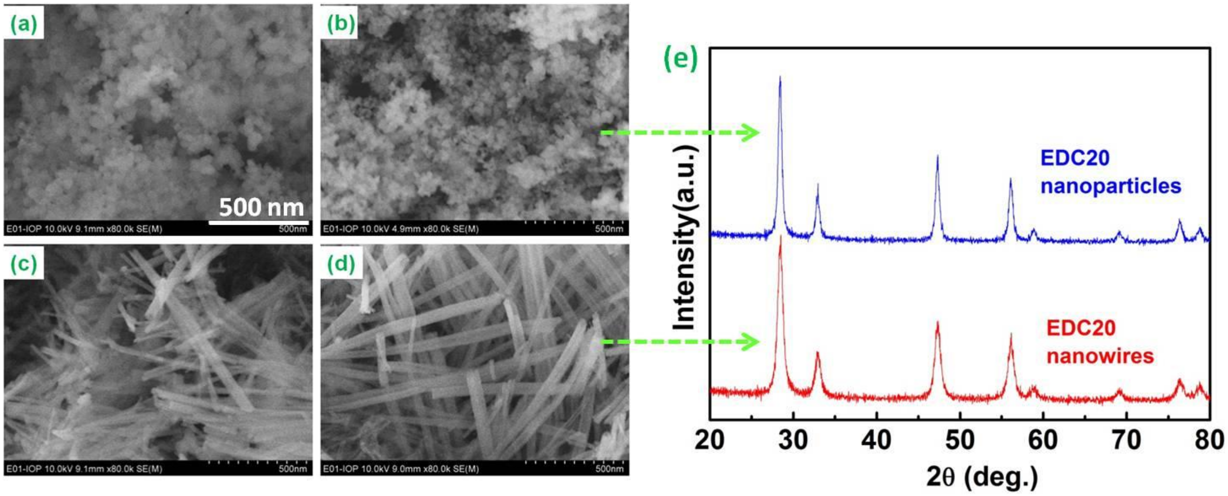

Figure S1 Morphology of EDC20 nanoparticle as co-precipitation for 1 minutes (a) and then calcinated at 700 ^oC in air (b); nanowire as co-precipitation for 1 hour (c) and then calcinated at 700 ^oC in air (d); the corresponding XRD patterns of calcinated EDC20 nanoparticle and nanowires (e).

The morphology of EDC is influenced by the aging time during co-precipitation. And it also influenced by the composition. Under the same synthesis condition, as the Eu doping level increases, the nanowire becomes narrower gradually. When the Eu content exceeds 20 mol.%, uniformed nanoparticles are synthesized. It indicates that with more Eu doing into CeO_2, the initial co-precipitated nanoparticles are not easy to agglomerate because of surface or lattice structure change. The morphology variation does not change the structure of CeO_2 with the same Eu doping content. For further study on the conductivity and catalytic activity, samples of nanowire morphology are selected.

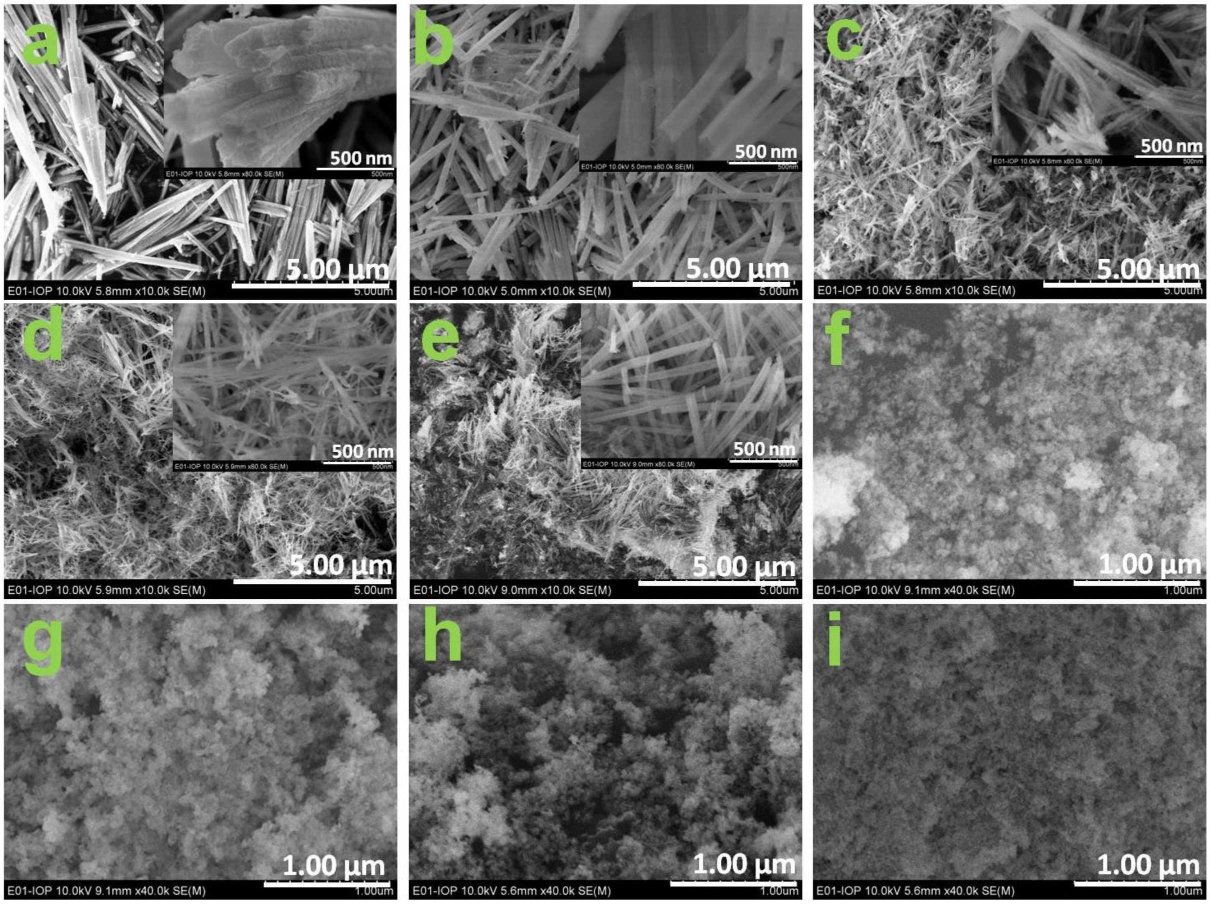

Figure S2 Morphology of CeO_2 (a), EDC5 (b), EDC10 (c), EDC15 (d), EDC20 (e), EDC25 (f), EDC30 (g), EDC35 (h) and EDC40 (i) nanomaterials synthesized via co-precipitation at 80 ^oC for 1 hour and calcinated at in air at 700 ^oC for 5 hours.

To indentify the contributions to the electrochemical impedance spectra, the dense (sintered at 1350°C for 10h) and nondense (sintered at 1200°C for 10h) ceramics of EDC15 samples were tested. Under the same conditions, the difference in impedance spectra would arise from grain boundary connection, excluding the influence of compositions. The typical impedance spectra of these samples

measured at 400°C in air are shown in Figure S2b. The spectra of the two samples both consist of two typical semicircles. The semicircles at higher frequency range, which are almost the same for the two samples, are ascribed to the contribution of bulk resistant. The semicircle at lower frequency range, which is relatively larger for the undensified ceramic, is ascribed to the contribution of grain boundary resistant. In the experiments, to compare conductivity of different samples and to eliminate the influence of grain boundary, all the pellets were sintered at 1350°C for 10h to get dense ceramic.

The conducting activation energy was calculated from the Arrhenius equation $\ln(\sigma T) = B - E_a/kT$. The bulk, grain boundary and total conductivity activation energy of different EDC samples are listed in Table S1.

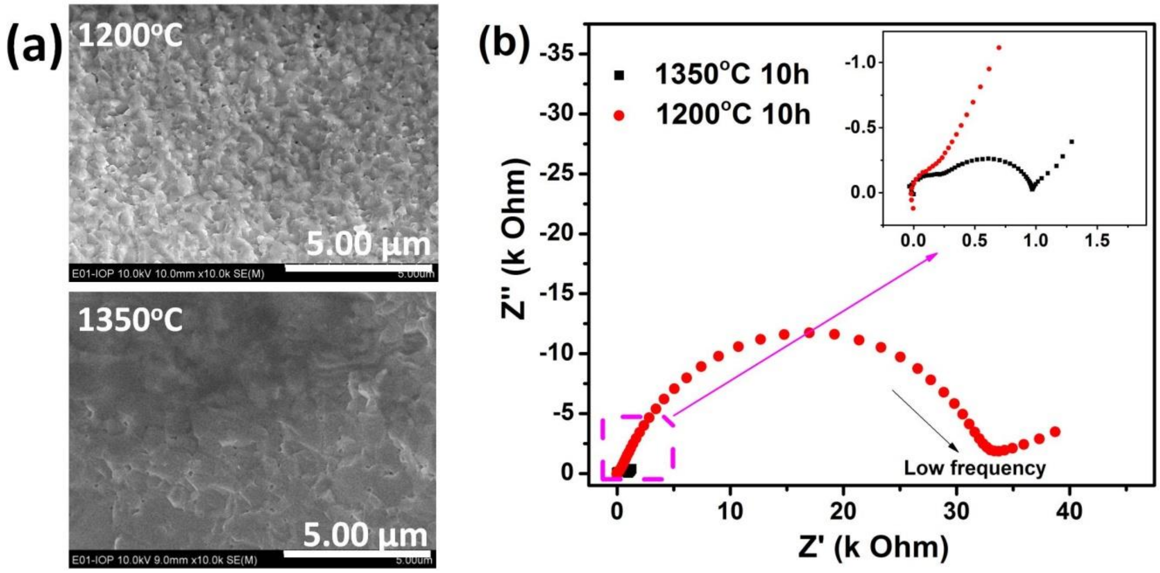

Figure S3 (Color online) Cross section of EDC15 pellet sintered at 1200 °C and 1350 °C in air, respectively (a). Impedance spectra measured at 400°C in air of EDC15 sintered in air at 1200°C and 1350°C for 10h, respectively. The insets are the magnified spectra corresponding to bulk resistance measure at high frequency. (b)

Table S1 Lattice Parameters of EDC samples

Sample	Lattice Parameter (Å)
CeO_2	5.411
EDC5	5.416
EDC10	5.420
EDC15	5.425
EDC20	5.428
EDC25	5.433
EDC30	5.436
EDC35	5.439
EDC40	5.442

Table S2 Conducting Activation Energy of Doped CeO_2 Samples

Sample	Conducting activation energy (eV)		
	Bulk	Grain boundary	total
EDC10	1.12	1.55	1.38
EDC15	0.92	1.55	1.37
EDC20	1.13	1.55	1.41
SDC20	1.01	1.42	1.31
